# Position Statement on Secukinumab in the Management of Plaque Psoriasis: The Malaysian Perspective

**DOI:** 10.1155/2019/8923168

**Published:** 2019-05-12

**Authors:** Asmah Johar, Suganthi Thevarajah, Agnes Heng, Lee Chin Chan, Chin Chwen Ch'ng, Najeeb Ahmad Mohd Safdar, Pubalan Muniandy, Tarita Taib, Wooi Chiang Tan, Kwee Eng Tey

**Affiliations:** ^1^Department of Dermatology, Hospital Kuala Lumpur, Malaysia; ^2^Agnes Heng Dermatology, Ipoh, Malaysia; ^3^Hospital Pulau Pinang, Georgetown, Malaysia; ^4^University Malaya Medical Center, Malaysia; ^5^Hospital Tuanku Ja'afar, Seremban, Malaysia; ^6^Sarawak General Hospital, Kuching, Malaysia; ^7^Department of Dermatology, Universiti Teknologi MARA, Shah Alam, Malaysia; ^8^Hospital Sultanah Aminah, Johor Bharu, Malaysia

## Abstract

Psoriasis is a chronic inflammatory skin disease affecting nearly 10% of dermatologic patients in Malaysia. Treatment options include topical agents and phototherapy as well as nonbiologic and biologic systemic therapy. Mild psoriasis can often be managed with topical agents. However, managing moderate to severe psoriasis is more challenging and may require systemic treatment with nonbiologics or biologics. Despite the availability of several biologics, there are many unmet clinical needs, which may be addressed by secukinumab, an IL-17A inhibitor. This position statement is based on an expert panel discussion and is intended to provide dermatologists an overview of existing options as well as to provide a better understanding of secukinumab and how it can be integrated into current practice. During the discussion, panel members examined current approaches and the role of secukinumab in plaque psoriasis management. Panel members estimated that up to 30% of patients have moderate to severe psoriasis but only 1-2% receive biologics. Highlights from the discussion were that (i) the threshold for biologic use should be lower, in line with international guidelines; (ii) studies have shown that secukinumab has several advantages over other biologics which are greater efficacy, sustained efficacy over time, rapid onset of action, and early evidence of possible disease-modifying potential; and (iii) ideal candidates for secukinumab are all patients of moderate to severe psoriasis, including those with history of treatment failure, difficult-to-treat patterns of psoriasis (nail, scalp, and palmoplantar psoriasis), psoriatic arthritis, and comorbidities and those aiming for clear skin. Panel members recommend that secukinumab be considered first line option among biologic therapies.

## 1. Introduction

Psoriasis is an immune-mediated and chronic systemic inflammatory disease affecting the skin and joints, with a large influence on patients' quality of life [1]. Among dermatological presentations in Malaysia, psoriasis affects approximately 10% of patients [2], and plaque psoriasis is the commonest form which is characterised by raised areas of sharply demarcated erythematous plaques [3]. It is thought to be caused by environmental triggers which activate inflammatory cells in the innate and adaptive immune pathways in genetically predisposed individuals. To address issues faced in treating this group of patients, an expert panel was convened by Novartis on May 13^th^, 2017, addressing current management as well as the potential role of secukinumab. Panel members included 11 clinicians in the field of dermatology from the Malaysian Ministry of Health, Ministry of Education, and private hospitals. The following article documents current evidence presented during the panel discussion and the opinion of panel members on psoriasis management.

Secukinumab is a monoclonal antibody and a first-in-class anti-IL-17A agent developed to target and block the action of IL-17A, an active protein in the inflammatory response that occurs in psoriasis [4]. Secukinumab has been available in Malaysia since 2016 [5] and is indicated in moderate to severe plaque psoriasis and active psoriatic arthritis as well as active ankylosing spondylitis [6, 7]. The recommended loading dose for treating psoriasis is 300mg by subcutaneous injection at weeks 0, 1, 2, 3, and 4, and treatment is continued as maintenance at the same dose every four weeks [8]. Clinical trial evidence has demonstrated the superiority of secukinumab over other biologics in the treatment of psoriasis by using the Psoriasis Area and Severity Index (PASI) as the primary endpoint. In the FIXTURE trial, comparing secukinumab (300mg and 150mg doses) to etanercept, the rate of patients achieving PASI 75 (75% reduction of PASI score from baseline) was higher in the secukinumab groups (77.1% in 300mg; 67% in 150mg) compared to etanercept (44%; p<0.001) [9]. Similarly, in the CLEAR trial, compared to ustekinumab, secukinumab was found to be more efficacious as assessed by achievement of PASI 90 (90% reduction of PASI score) at week 16 (79.0% vs 57.6%; p<0.0001) [10].

The World Health Organization reports that worldwide prevalence of psoriasis ranges between 0.09% and 11.4% [11]. This prevalence is observed to be higher in Caucasians compared to other populations [12, 13]. In Malaysia, the national prevalence is unknown as there has been no population-based epidemiological study on psoriasis [3]. However, there have been smaller studies which provide some insight about its prevalence among patients with skin-related diagnoses. A study by Sinniah et al., performed among 5,607 outpatients attending the dermatology clinic at Hospital Tengku Ampuan Rahimah, Klang, between 2003 and 2005, found that 9.5% had a diagnosis of psoriasis [2]. In addition, the Malaysian Psoriasis Registry, which includes only patients with psoriasis, reported a total of 12,615 patients between 2007 and 2014. The majority of patients recorded were Malays (50.7%), followed by Chinese (21.8%), Indians (18.2%), other ethnic groups (9.1%), and Orang Asli (0.1%) [14]. Registry data is collected from 23 dermatology clinics in 21 government hospitals (including two academic centres) and two private hospitals and it is important to bear in mind that the registry does not include all Malaysian patients with psoriasis. Overall, despite the lack of nationally representative studies, it may be reasonable to suppose that the national prevalence would be similar to other countries in Asia perhaps in the range of 0.2 to 0.5% [15–17].

## 2. Management of Psoriasis

### 2.1. Available Psoriasis Treatment

The approach to psoriasis treatment depends on the clinical assessment of severity. These include Psoriasis Area and Severity Index (PASI), Physician Global Assessment (PGA), and Dermatology Life Quality Index (DLQI). The Malaysian Clinical Practice Guideline (CPG) recommends treating mild psoriasis with topical therapy first, and mild psoriasis is often treatable this way [3]. Unlike mild psoriasis, moderate to severe psoriasis may need to move beyond topical agents and on to phototherapy, systemic therapy, or a combination of both [3, 18, 19]. Phototherapy such as ultraviolet B (UVB) and psoralen with local ultraviolet A (PUVA) may be given 2 or 3 times a week. Nonbiologic therapy, on the other hand, is given when phototherapy is not effective or when the disease has a significant impact on the patient's quality of life whether or not the disease is localised or covering a large area. As always, preference of the patient should be taken into consideration [3]. When systemic therapy is being considered, there are two options: nonbiologics or biologics. Nonbiologics that can be considered are methotrexate, acitretin, and cyclosporine. Current options among biologics are ustekinumab, adalimumab, etanercept, and infliximab. Secukinumab is the latest addition to the armamentarium.

## 3. Treatment Landscape of Moderate to Severe Psoriasis in Malaysia

As estimated by the majority of panel members, about 21-30% of psoriasis patients have moderate to severe disease and they are often treated with systemic therapy rather than phototherapy [14]. Most patients are unable to come for phototherapy due to logistical, time, and work issues. According to the Malaysian Psoriasis Registry, only 3.5% of adult psoriasis patients undergo phototherapy while 19.4% receive systemic therapy whether it is nonbiologic or biologic therapy [14]. The majority of patients receiving systemic therapy are on nonbiologics, which are most commonly, methotrexate (71.5%), acitretin (19.9%), sulphasalazine (5.7%), and cyclosporine (4.1%) [14]. Only a small proportion of these patients receive biologic agents (2.4%), which was similar to clinical experience of the panel members (1-2%). Several biologics have been approved and are available in Malaysia including infliximab, etanercept, adalimumab, ustekinumab, and, more recently, secukinumab. However, despite the availability of biologics, the number of patients receiving them is very low and this may indicate that a large proportion of patients are not receiving optimal treatment.

### 3.1. Current Role of Biologic Therapy

Panel members believe that biologic therapy should be used earlier in the management of psoriasis than currently recommended in Malaysia [3]. Current local recommendations are that biologic therapy should be used only in severe psoriasis (PASI score >20, DLQI >20 or BSA >30%) in situations where treatment has failed or is contraindicated or the patient is intolerant to nonbiologics [3]. In comparison, the panel noted that international guidelines such as the British Association of Dermatologists (BAD), National Institute for Health and Care Excellence (NICE), and American Academy of Dermatology (AAD) guidelines recommend lower thresholds for consideration of a biologic therapy [18–21]. A patient with a PASI score of >10 or BSA of >10% and DLQI score of >10 is eligible for biologic therapy according to BAD and NICE guidelines [18, 21]. Meanwhile the AAD's threshold is even lower, BSA ≥5% [19, 20]. Moreover, the guidelines also state that biologics may be given earlier when the disease affects difficult-to-treat areas such as hands, feet, and facial or genital regions [19, 21]. From the patient's perspective, the psychosocial impact of psoriasis might be underestimated when based on clinical assessment. Overall, panel members felt that international thresholds would be more appropriate and therefore thresholds should be lower especially when the patients' perspective is taken into consideration.

## 4. Role of Secukinumab in Moderate to Severe Psoriasis Management

Despite the availability of various other biologic agents, prior to secukinumab, there were several unmet needs. These were issues surrounding low rates of clear skin achievement, diminishing biologic efficacy over time, and slow onset of action as well as several safety concerns. In clinical trials of earlier biologics, efficacy had been based on the current standard treatment response of PASI 75 [22]. However, in terms of achieving clear skin (PASI 90 and 100), the percentage of achievement by earlier biologics has been found to be relatively low [21]. Also, some biologics tend to exhibit diminishing efficacy over time due to immune reaction against the biologics in some patients. This was observed in several biologics including anti-TNF and anti-IL-12/23 agents [23–26]. The majority of panel members either “strongly agreed” or “agreed” that this is an important concern. Next is the issue that some biologics take longer to produce the desired outcome. A systematic review by Nast et al. showed that time taken to achieve PASI 75 in 25% of study populations varied between biologics: etanercept (6.6 weeks), adalimumab (4.6 weeks), and ustekinumab (4.6 weeks). Generally, most took more than four weeks to achieve PASI 75 [27]. Panel members also raised concerns about safety data reported in clinical trials, particularly, reactivation of tuberculosis, rates of infections, and allergic reactions [28].

### 4.1. Secukinumab Superiority Compared to Earlier Biologic Agents

Clinical trial evidence for secukinumab addresses the limitations of earlier biologic therapies. Panel members acknowledged that the trial data was consistent with their own experience in practice. Firstly, as mentioned earlier, data from the FIXTURE and CLEAR trials showed secukinumab superiority over other biologics in terms of achieving PASI 75. Both studies also reported higher rates of nearly clear/clear skin (PASI 90/100) in the secukinumab group against its respective comparator groups. The proportions of patients in the FIXTURE study achieving PASI 90 (54.2% vs 20.7%) and PASI 100 (24.1% vs 4.3%) were significantly higher at week 12 in the secukinumab group compared to etanercept (p<0.001) [9]. In the CLEAR study, secukinumab achieved a higher rate of PASI 90 (79.0%) compared to ustekinumab (57.6%; p<0.0001) at week 16 and the rate was sustained over a period of 52 weeks [29].

Secondly, unlike other biologics, secukinumab has the ability to sustain efficacy over time [23–26]. Diminishing efficacy with biologics could occur when patients are undergoing initial treatment (primary failure) or during a subsequent treatment following a relapse (secondary failure) [26]. The most likely explanation for this is the immunogenic response to a biologic which causes the formation of anti-drug antibodies (ADAs) which increase drug clearance and may neutralize product binding capacity [23–26]. Secukinumab has been demonstrated to have low immunogenicity and prevalence of detected ADAs [30, 31]. Furthermore, despite the detection of ADAs, secukinumab trials did not show a decrease in efficacy [9, 30, 32]. Relatedly, an analysis of the SCULPTURE study confirmed the long-term efficacy and safety data of secukinumab [33]. Secukinumab treatment showed sustained efficacy of PASI 75, 90, and 100 response rates from Year 1 (88.9%, 68.5%, and 43.8%, respectively) to Year 5 (88.5%, 66.4%, and 41%, respectively) (Table 1). In addition, secukinumab also demonstrated sustained absolute PASI rates (PASI ≤1, ≤2, ≤3) from Year 1 (58.6%, 67.9%, and 74.1%, respectively) to Year 5 (53.3%, 66.4%, and 75.4%, respectively) [33].

Secukinumab also addresses the slow onset of action and safety concerns about reactivation of tuberculosis. Secukinumab has a more rapid onset of action compared to etanercept and ustekinumab. In the FIXTURE study, secukinumab 300mg took a shorter duration (3 weeks) compared to etanercept (7 weeks) to achieve 50% reduction of mean PASI score [9]. Meanwhile, the CLEAR study demonstrated significantly earlier onset compared to ustekinumab, achieving PASI 75 as early as week 1 (p<0.05) [29]. Even though the general safety profile of secukinumab is comparable to other biologics, secukinumab has been shown to have no tuberculosis reactivation [28]. Two pooled analyses, by Tsai et al. and Kammuller et al., reported that secukinumab showed no evidence for reactivation of previous or latent tuberculosis infection [34, 35]. Long-term SCULPTURE study data showed no increase in annual adverse event rates throughout the five years of treatment [33].

**Figure 1:**
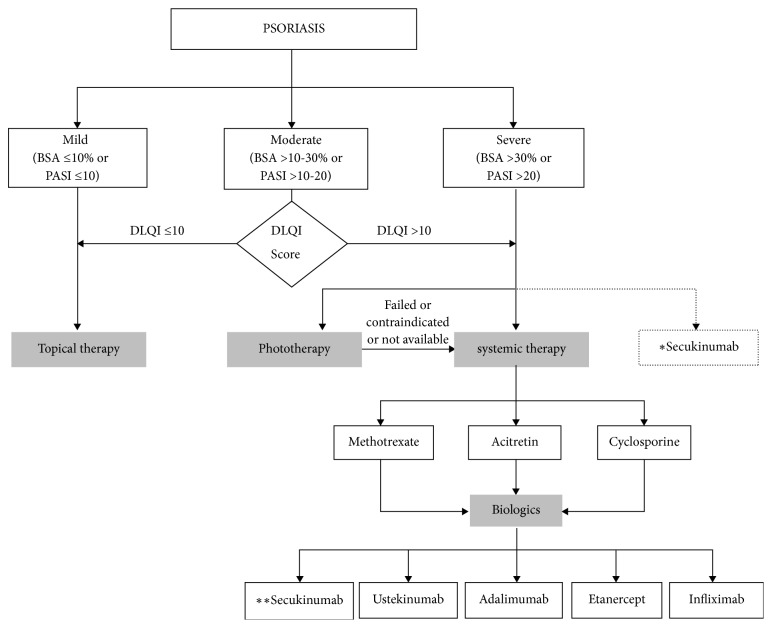
*Psoriasis treatment flowchart and role of secukinumab according to panel members' consensus*. ^*∗*^Secukinumab position as per indication in prescribing information. ^*∗∗*^Secukinumab position as suggested by panel members (first line option among biologic therapies).

It was further discussed that aside from the above advantages, secukinumab has recently been shown to have the potential for disease-modifying effect on moderate to severe psoriasis. Relapse and exacerbation tend to be common after discontinuation of any treatment or sometimes even while the patient is still on treatment [36]. With secukinumab, a recent study by Lebwohl et al., which followed moderate to severe psoriasis patients after discontinuation of 1-year treatment (n=120), reported that 21% of patients on secukinumab 300mg did not relapse after 12 months of discontinuation and 10% of them remained relapse free even after 24 months of discontinuation [37]. This potential is still being studied, and there is an ongoing trial examining the disease-modifying potential of secukinumab in new-onset moderate to severe psoriasis [38]. Most panel members agreed that the impact of this potential is a key consideration with 80% considering it “very important” and 20% considering it “absolutely essential”. Given the strong comparative evidence for secukinumab over other biologics, all panel members either “strongly agreed” or “agreed” that it should be considered as the first line option among biologic therapies. It was however noted that as per the prescribing information, secukinumab is indicated for the treatment of moderate to severe plaque psoriasis in adult patients who are candidates for systemic therapy or phototherapy [8] and it may be used as first line systemic therapy (Figure 1).

### 4.2. Ideal Patient Profile

As indicated in the drug prescribing information, all patients with moderate to severe psoriasis who are candidates for phototherapy or systemic therapy are eligible for secukinumab [8]. Besides using secukinumab as a first line option, panel members identified several types of patients who may be ideal candidates for secukinumab. The first patient type includes individuals who have experienced treatment failure, whether with phototherapy or nonbiologic or other biologic therapy [3]. Studies with earlier biologics reported that patients with prior exposure to biologics tend to have lower response to other biologics, specifically, etanercept [39] and ustekinumab [40]. On the other hand, secukinumab has demonstrated skin improvement despite prior biologic treatment failure. In a pooled analysis by Griffith et al., secukinumab had a greater percentage of patients achieving PASI 90 (90% reduction in PASI score from baseline) among patients with history of biologic failure compared to etanercept (42% vs 12.5%; p<0.05) [41]. Similarly, in the CLEAR study, secukinumab showed a superior PASI 90 response at week 52 (76% vs 61%; p<0.0001) compared to ustekinumab regardless of previous exposure to treatment [29].

A second type of ideal candidates comprises those with difficult-to-treat patterns of psoriasis such as when the nail, scalp, and palmoplantar areas are affected. Nail psoriasis is a common feature of psoriasis and leads to a notable impairment in quality of life. Although there are many recent improvements in the treatment of psoriasis, the options for nail psoriasis are limited. An open-label retrospective study reported clinically significant improvement in nail psoriasis measured by NAPSI in patients treated with biological therapies (adalimumab, etanercept, infliximab, and ustekinumab). The mean NAPSI decreased from 32.01 at week 0 to 12.37 at week 12 [42]. A Cochrane review reported that infliximab 5 mg/kg showed 57.2% nail score improvement versus -4.1% for placebo (P < 0.001); golimumab 50 mg showed 33% improvement versus 0% for placebo (P < 0.001), both after medium-term treatment (6 to 12 months) [43]. The TRANSFIGURE study demonstrated that secukinumab provides significant improvement in nail psoriasis with greater change in the Nail Psoriasis Severity Index (NAPSI) compared to placebo (-45.3% vs -10.8% at week 16; p<0.0001) [44]. Meanwhile, in the SCALP study, secukinumab demonstrated greater benefit in scalp psoriasis as measured by percentage of patients achieving Psoriasis Scalp Severity Index (PSSI) 90 against placebo (52.9% vs 2.0% at week 12; p<0.001) [45]. Related to palmoplantar psoriasis, the GESTURE and 2PRECISE trials demonstrated greater improvements than placebo [46, 47]. In GESTURE, Gottlieb et al. reported that the percentage of patients who achieved Palmoplantar Investigator's Global Assessment (ppIGA) 0 (clear) or 1 (almost clear) was significantly higher compared to placebo (33.3% vs 1.5% at week 16; p<0.0001) [46], whereas in the 2PRECISE study, Mrowietz et al. evaluated percentage of patients achieving Pustular Palmoplantar PASI (ppPASI) 75 comparing between secukinumab and placebo groups. At week 16, a higher proportion of patients receiving secukinumab 300mg and 150mg (26.6% and 17.5%, respectively) achieved the desired outcome compared to patients receiving placebo (14.1%) [47].

Patients with psoriatic arthritis (PsA) are also ideal candidates for secukinumab, especially PsA phenotype individuals with joint involvement who tend to have a poorer prognosis [48]. In the FUTURE 2 trial, the rate of subjects achieving an American College of Rheumatology (ACR) score of 20 in the secukinumab group was significantly higher compared to placebo (54% vs 15%; p<0.0001) [49]. Even though head-to-head data comparing secukinumab against other biologics is not yet available, a study by Nash et al. indirectly compared efficacy of secukinumab with adalimumab using pooled data from the FUTURE 1 and 2 studies (secukinumab 150mg) and ADEPT study (adalimumab 40mg) [50]. The study found that secukinumab was associated with higher ACR 20 (72.2% vs 56.3% at week 48; p=0.01) and ACR 50 (45.9% vs 32.5% at week 16; p=0.029) response rates compared to adalimumab [50]. Meanwhile, in a network meta-analysis by McInnes et al., secukinumab was shown to be significantly more efficacious among PsA patients in achieving ACR 20, 50, and 70 compared to ustekinumab [51].

Psoriasis patients at risk of tuberculosis may also be ideal candidates of secukinumab as there has been no evidence of latent tuberculosis reactivation. Kammüller et al. performed a pooled analysis of five randomized controlled trials (N=2,044) involving 132 patients with a history of pulmonary and positive latent tuberculosis reported that there were no cases of tuberculosis reactivation among the subjects [35]. Therefore, patients with history of tuberculosis are suitable secukinumab candidates. This is notable since TNF inhibitor treatment has been shown be associated with higher incidence of tuberculosis reactivation [52–56]. This may be important to consider since there have been concerns about tuberculosis infection rates in Malaysia [57, 58]. However, secukinumab should not be given to patients with active tuberculosis. Antituberculosis therapy should be considered prior to initiation of secukinumab in patients with latent tuberculosis.

Lastly, another ideal patient type includes individuals who need to achieve clear skin in a short time or are highly motivated by quality of life or lifestyle factors. As discussed earlier, studies have demonstrated that a higher proportion of patients achieve PASI 90 and 100 with secukinumab compared to other biologics [9, 29]. An example discussed by panel members was that of a young woman who is to get married soon and wants to look her best for her wedding. In such cases, secukinumab may be the most appropriate treatment option.

## 5. Monitoring of Patients on Secukinumab

### 5.1. Monitoring Frequency

Panel members differed somewhat on the duration of therapy and appropriate frequency of follow-up monitoring as well as the minimal treatment target. Approximately half the panel members would prescribe secukinumab for more than 24 months. On frequency of monitoring tests, the majority of panel members (40%) felt that patients should be monitored every 3 months, followed by 30% who would monitor every 4-6 months. However, the frequency of monitoring should be further based on disease severity, associated comorbidities, and presence of adverse reactions. At each visit, treatment response assessment is required and should be based on minimal treatment target. As recommended for biologic therapies in general, clinical examination and investigations are required prior to and during treatment to monitor for common side effects [28].

### 5.2. Safety

The most common adverse event of secukinumab is nasopharyngitis. A pooled analysis of secukinumab clinical trials by van de Kerkhof et al. found that around 27% of patients who received secukinumab administration experienced nonserious, mild, or moderate nasopharyngitis. Next most common adverse events were headache (10%) and upper respiratory tract infection (8%). Candida infections were rare and easily manageable. They were usually mild, resolved spontaneously, or responded to standard treatment. Besides, most cases with neutropenia were also mild, transient, and reversible. Serious adverse events such as gastrointestinal or central nervous system disorders occurred at low incidences across treatments. No death occurred during the treatment with secukinumab [59]. Secukinumab is shown to have no increased risk of inflammatory bowel syndrome such as Crohn's disease (CD) and ulcerative colitis (UC). A study by Deodhar et al. analysed data from ten phase II and phase III studies evaluating secukinumab in psoriasis patients (N=3,430). Results showed that CD or UC were infrequently reported among the study populations (incidence rates of Crohn's and inflammatory bowel disease were 0.11% and 0.15%, respectively) and neither condition was dose-dependent. Exposure Adjusted Incidence Rates (EAIR) of CD and UC observed in secukinumab-treated patients were consistent with those reported in the literature in psoriasis, psoriatic arthritis, and ankylosing spondylitis populations [60]. In conclusion, secukinumab exhibited a favourable safety profile in all pivotal phase III trials. There is currently no safety data on secukinumab use among pregnant women.

### 5.3. Treatment Response

The Malaysian CPG's minimal target is either PASI ≥75 or PASI 50 to PASI 75 plus DLQI ≤5 [3], whereas BAD recommends the same PASI reduction but a lower DLQI score (DLQI ≤4) [21]. However, as patients seem to respond very well to biologics, panel members discussed the possibility of increasing the minimum target to PASI 90 or 100. Panel members agreed that from the patient's perspective, the ultimate goal would be obtaining complete skin clearance causing no or minimal impact on quality of life. Strober et al., in his study examining the effect of total skin clearance on skin-specific symptoms and QoL in patients with moderate to severe plaque psoriasis, showed that patients who achieved PASI 100 had lower DLQI scores compared to patients who achieved PASI 75 to <100 [61]. Viswanathan et al. reported similar results in that subjects with “total skin clearance” had no impairment in their quality of life compared to subjects with “almost clear” skin [62]. Although the research evidence on this topic is still limited, panel members believe that reaching PASI 90 or PASI 100 is clinically achievable.

## 6. Conclusion

This position statement was developed to convey opinions and insights from a dermatology expert panel about current treatment approaches and the role of secukinumab. Several unmet needs related to existing biologics were identified including low rate of clear skin achievement, diminishing efficacy, delayed onset of action, and safety concerns especially tuberculosis reactivation. Among the recommendations made by the panel were that the threshold for considering biologic therapy should be lowered to allow earlier use of biologics. On the role of secukinumab, panel members agreed that secukinumab should be placed as first line option among biologics in moderate to severe psoriasis. This was supported by clinical trial evidence which demonstrated secukinumab advantages in addressing current treatment gaps and that it should be considered among all patients with moderate to severe psoriasis with or without psoriatic arthritis.

## Figures and Tables

**Table 1:** Dosing schedule and efficacy of secukinumab.

Types of biologics	Dosing schedule [33]	Expected onset of clinical effect (week) [33]	Review of response (week) [33]	Efficacy at week 10 to 16 (PASI 75) [32]	Long-term efficacy [33]
Secukinumab	Subcutaneous, 300mg at week 0, 1, 2, 3, 4 and then every 4 weeks	1	12	90.1%	PASI 75: 88.5%
					PASI 90: 66.4%
					PASI 100: 41.0%
